# Burnout and Its Relationships with Alexithymia, Stress, and Social Support among Romanian Medical Students: A Cross-Sectional Study

**DOI:** 10.3390/ijerph14060560

**Published:** 2017-05-25

**Authors:** Ovidiu Popa-Velea, Liliana Diaconescu, Alexandra Mihăilescu, Mara Jidveian Popescu, George Macarie

**Affiliations:** Department of Medical Psychology, Faculty of Medicine, University of Medicine and Pharmacy “Carol Davila”, 050474 Bucharest, Romania; idiac2002@yahoo.com (L.D.); andrast79@yahoo.com (A.M.); mara.jidveian@gmail.com (M.J.P.); george.macarie@mail.com (G.M.)

**Keywords:** medical students, burnout, perceived stress, social support, alexithymia, gender

## Abstract

Medical school students often experience emotional difficulties when handling the challenges of their formation, occasionally leading to burnout. In this study, we measured the prevalence of burnout and its relationships with perceived stress, perceived social support, and alexithymia in medical students from the largest academic medical community in Romania. A cross-sectional survey was administered to a randomized sample of 299 preclinical medical students at the University of Medicine in Bucharest. Participants completed four standardized questionnaires. In addition to the assessment of burnout prevalence, stepwise backward regression was used to establish which variables had the highest correlation to burnout components. Further, *t*-tests were run to assess gender-related differences. Overall, burnout prevalence was 15.05%. Perceived stress was found to be the strongest predictor of emotional exhaustion and lack of accomplishment, while the strongest predictors of depersonalization were low perceived social support (in women) and alexithymia (in men). Women appear to be more vulnerable to two of the components of burnout (emotional exhaustion and low personal accomplishment) and associate higher perceived stress and alexithymia. These results suggest that interventions addressing academic burnout could benefit from being gender-specific, with focus on key elements, such as perceived stress and alexithymia.

## 1. Introduction

Medical education is considered to be one of the most demanding fields of specialized training, in terms of program length and emotional requirements [1,2], particularly during the preclinical years [3,4]. The distress experienced during this period has consistently been reported [5,6,7] and has gained constant attention in recent decades [8]. A significant outcome of the cumulative distress experienced throughout the academic track [1,9] is represented by the burnout syndrome, defined as the presence of emotional exhaustion, depersonalization, and impaired personal accomplishment, following repeated exposure to workplace stressors [10,11]. In medical academic environments, even though the prevalence of burnout ranges widely [8,12,13,14,15], it is consistently referred to as a worrisome issue, because of its consequences [15,16,17]. At the individual level, burnout is often associated with poor health [14], alcohol, drug abuse [18], and an increased risk of suicidal ideation [12], all of which can in turn impair professional life via increased risk of dropping out [19], cynicism, low empathy [20], and a global deterioration of job performance. Numerous studies suggest that the high prevalence of burnout in physicians may often originate in medical school [3,13,14,15,19,21,22,23], including during the preclinical years [24,25].

The causes of burnout in medical students are often described in literature as having an overwhelming association with specific characteristics of the academic environment, such as the significant demands of the medical educational process, school schedule, the long duration of studies [1], the vast nature of the curriculum and its potentially “hidden content” [26], frequent examinations [27], lack of control [8], massive workload [28], and the difficulties in adapting to a new social sphere.

Still, literature data [29,30,31] consistently suggest that personal factors could also have a decisive importance on the onset of burnout, as they can moderate and alleviate the challenges of integrating into a changing social environment. These often underexplored factors could be used as a potentially significant resource in the prevention of burnout, especially when the social determinants of burnout are unreachable or unchangeable. In this sense, alexithymia, perceived social support, and perceived stress are all remarkable, because they share the property of being a reflection of the individual’s position in relation to the surrounding circumstances. Additionally, these factors have been previously described in the literature as potentially related to the onset and outcome of the burnout syndrome:

- Alexithymia is characterized by (a) difficulty identifying one’s feelings and distinguishing them from bodily sensations and (b) difficulty communicating one’s emotions to others [32,33]. Its prevalence rises up to 17% in some reports [34,35,36], and it seems to be positively correlated with the perception of subjective distress [34,37] and depression [38]. In the general population, alexithymia is associated with higher global scores of burnout [38,39], or at least with some of its components (e.g., depersonalization [40]). Moreover, this relationship has also been observed in students [41,42]. In particular, medical students may be more susceptible to alexithymia, given their lack of adjustment to the challenging situations encountered in their training.
- Perceived stress (along with lower levels of social support) has been described as being more prevalent in academic contexts when compared to the general population [7] and being related to higher levels of overall burnout or its components [8,13,14,17,43]. Generally, in the context of professional life, it is often associated with a loss of interest for work, difficulties in interpersonal relationships, and a poor physical condition [16,44].
- Perceived social support is reported to represent a protective factor against stressors [45,46], for both youngsters [47] and students [48,49,50], while a lack thereof may be a risk factor for burnout [13,14]. These findings, in conjunction with the fact that medical students report greater needs of support than students from other academic domains [49], make this parameter important to examine, in connection to burnout and perceived stress.

Although each of the three abovementioned variables seems to be individually related to burnout (or at least to the predisposition towards it), little is known about their comparative importance, particularly in academic environments. Consequently, research focused on describing their associations to burnout appears relevant, especially since (a) students may be vulnerable to the cumulative effects of burnout throughout their training [1,2,3,15] and (b) finding these relationships may provide targets for a potential intervention, either specific (individual or group psychotherapy) or more general (e.g., institutional strategies).

The current study aims to examine (a) the prevalence of academic burnout in Romanian medical students during their preclinical years of training and (b) the comparative impact of perceived stress, perceived social support, and alexithymia on the burnout scores of these subjects. Additionally, our analysis is oriented towards identifying the possible role that gender plays in regard to these associations. We hypothesized that the prevalence of academic burnout in Romanian medical students is within ranges reported in current literature [8,12,13,14,15] and that perceived stress, alexithymia and perceived social support contribute to the scores of burnout, with a distinctive influence of gender.

## 2. Materials and Methods

### 2.1. Participants

Participants were undergraduate students undergoing their preclinical training at the University of Medicine and Pharmacy “Carol Davila” Bucharest, Romania (UCD). We focused on this study group, since this university is the largest medical school in Romania, gathering students from all regions of the country. Four hundred students were randomly selected from a total of 2000, with a class of 12–15 students as the primary selection unit. Finally, 299 students (94 men, 205 women; mean age = 19.23, SD = 0.59) agreed to participate in the study (74.75% response rate). All participants met the inclusion criteria, set as being at least 18 years of age, having no self-reported psychiatric morbidity, cognitive deficits or any other impairments which would render the understanding and completion of the study questionnaires difficult.

Almost 70% of the participants were women, which is consistent with the higher proportion of female enrollment in Romanian medical schools. The sex ratio between men and women in our sample (94/205 = 1:2.18) was not significantly different from the sex ratio of all students (1:1.99) (χ^2^ = 0.546, *p* = 0.461, ns).

### 2.2. Procedure

The study was performed in 2015 and 2016, at the beginning of the second school semester. All participants completed informed consent forms before they took part in the study. The study was run in accordance with the World Medical Association Declaration of Helsinki and with the ethical guidelines published by the Committee of Ethics at the UCD.

The participants received sealable envelopes containing an explanatory statement, the written consent form and the questionnaires. They were instructed to return the completed questionnaires, together with the written consent form in the sealable envelope. The responses were processed anonymously and a numerical code was assigned for each participant. The collected data were accessible exclusively to study researchers (LD, AM, and MJP). Regular didactic staff was not present during the distribution, collection, or interpretation of questionnaires.

### 2.3. Measurement Tools

All participants received four questionnaires: Maslach Burnout Inventory [10], Toronto Alexithymia Scale [51], Perceived Stress Scale [52], and Duke-UNC Functional Social Support Questionnaire [53]. The rationale for choosing them was based on the following existing data:

(1) Maslach’s 22-item Burnout Inventory (MBI) is a widely used tool to assess burnout. It contains three subscales, reflecting one’s personal feelings and attitudes: emotional exhaustion (EE) (9 items), depersonalization (DP) (5 items), and lack of personal accomplishment (LPA) (8 items). The answers can range from 0 (never) to 6 (every day), with each subscale score computed through summation. The test is generally easy to perform and has a good reported test–retest reliability of up to 0.82 and high validity [54,55]. In our study, we considered the criteria of burnout to be met if the participants had a score above 30 on the EE scale and if at least one of the scores of the other two dimensions were above the recommended cut-off points (DP: 11; LPA: 35).

(2) TAS-20 is currently considered to be one of the most valid measures of alexithymia [56]. It is a self-report scale with 20 items, on which scores higher than 61 indicate alexithymic characteristics. This test has been reported to have good internal consistency (Cronbach’s alpha = 0.81) and test–retest reliability (0.77, *p* < 0.01) [51], as well as adequate levels of convergent and concurrent validity.

(3) Perceived Stress Scale (PSS) is a 14-item self-report instrument, designed to measure the degree to which situations in one’s life are appraised as stressful. Each item is rated using a 5-point Likert type scales, the total of which may range between 0 and 56. This psychometric tool has been described to depict convergent validity, indicated by its solid relationships with depressive (*r* = 0.76, *n* = 332) and physical (*r* = 0.70, *n* = 64) symptomatology scales [52].

(4) The Duke-UNC Functional Social Support Questionnaire (FSSQ) measures the strength of the person’s social support network. It consists of 8 items, scored on a 1 to 5 scale, with higher scores corresponding to a greater perceived social support. This instrument has been reported to have high construct validity and high internal reliability (Cronbach’s alpha = 0.81–0.92) [53].

### 2.4. Design and Analysis

This cross-sectional study measured burnout, alexithymia, perceived stress, and perceived social support, with burnout as a dependent variable, and the other three as independent variables. Responses were processed using the SPSS Statistics 17.0.1 software package (SPSS, Inc., Chicago, IL, USA). The analysis included firstly a descriptive level, focused on assessing where our study variables lie in the normality–abnormality continuum. Next, a series of *t*-tests for independent samples were run to assess gender-related differences and their significance. Later, stepwise backward regression analysis was used to establish the strength of associations between variables. This analysis was also run separately by gender. Missing data were handled through listwise deletion. Assumptions of data normality and linearity were confirmed using histograms and partial plots. The independence of errors was checked through the Durbin–Watson test and homoscedasticity using *ZRESID and *ZPRED plots. For all calculations, the threshold of statistical significance was *p* < 0.05.

## 3. Results

### 3.1. Descriptive Analysis

15.05% of the participants met all criteria for burnout. Emotional exhaustion was encountered in 16.72% of study participants (10.63% men, 19.51% women), depersonalization in 28.42% (men: 39.36%, women: 23.41%), and lack of personal accomplishment in 10.70% (men: 11.70%, women: 10.24%). Total prevalence of clinical alexithymia was 6.02% (men: 2.12%, women: 7.80%). The percentage of perceived stress above the 3rd quartile was 26.42% (men: 15.95%, women: 31.22%). The percentage of respondents with perceived social support below the 1st quartile was 25.41% (men: 25.53%, women: 25.36%).

### 3.2. Gender Differences

Women had higher mean values for two out of the three components of burnout (emotional exhaustion and lack of personal accomplishment) but also for alexithymia and perceived stress. Significantly lower means were found in men at emotional exhaustion (t = −3.219, *p* < 0.001), alexithymia (t = −0.414, *p* < 0.016), and perceived stress (t = −3.065, *p* < 0.002) (Table 1).

### 3.3. Burnout and Its Associations

First, we ran separate Pearson’s correlations between the three components of burnout (EE, DP, and LPA) and perceived stress, alexithymia, and perceived social support, respectively. These correlations were statistically significant, suggesting that the studied dimensions were not confounding (Table 2).

Next, we ran a hierarchical multiple regression (backward method), in order to assess the weight of each contributing factor on the scores of the burnout components, as recommended by the author of the questionnaire [57]. Using this procedure, all independent variables (alexithymia, perceived stress, and perceived social support) were introduced into one model and the non-significant relationships among them were eliminated, resulting in a final model that ensured a minimum number of significant predictors.

For emotional exhaustion in men, the only significant predictor was perceived stress (*p* < 0.005), whereas in women the significant predictors were perceived stress (*p* < 0.0005) and alexithymia (*p* < 0.030). In men, regression eliminated, at the first step, perceived social support (coefficient = 0.002, 95% confidence interval [CI] = −0.222 ÷ 0.226) and, at the second step, alexithymia (coefficient = −0.116, 95% CI = −0.302 ÷ 0.070). In women, regression eliminated, at the first step, perceived social support (coefficient = −0.117, 95%, CI = −0.301 ÷ 0.067).

Concerning depersonalization, the significant predictors among men were perceived stress (*p* < 0.029) and alexithymia (*p* < 0.028), while for women the only predictor was perceived social support (*p* < 0.020). In men, regression eliminated, at the first step, perceived social support (coefficient = −0.165, 95% CI = −0.349 ÷ 0.018); for women, regression eliminated, at the first step, alexithymia (coefficient = 0.021, 95% CI = −0.073 ÷ 0.115) and, at the second step, perceived stress (coefficient = 0.070, 95% CI = −0.074 ÷ 0.214).

On the scale describing lack of personal accomplishment, the only significant predictor in men was perceived stress (*p* < 0.0005), whereas for women it was perceived stress (*p* < 0.0005) and alexithymia (*p* < 0.003). In men, regression eliminated, at the first step, perceived social support (coefficient = 0.057, 95% CI = −0.160 ÷ 0.275) and, at the second step, alexithymia (coefficient = −0.116, 95% CI = −0.302 ÷ 0.070). In women, regression eliminated, at the first step, perceived social support (coefficient = −0.081, 95% CI = −0.244 ÷ 0.083).

The detailed results are displayed in Table 3.

## 4. Discussion

This study examined the prevalence of burnout and its relationships to alexithymia, perceived stress, and perceived social support among Romanian undergraduate medical students.

We found the prevalence of burnout in our sample to be 15.05%, which is at the lower end among rates reported in other publications concerning this issue [8,12,13,14,15]. Among the individual burnout components, depersonalization was met in the highest percentage of participants (both men and women), suggesting that counseling strategies should focus on this dimension, for example, through exploring the individual discrepancies between expectations regarding medical school and its demands.

Among the three studied variables, perceived stress was found to exhibit the strongest association with burnout, in all of its components, and to single handedly explain 10–24% of the variance in burnout. This finding is sustained by other literature data that report a solid correlation between perceived stress and various components of burnout [13,16,31,58,59]. The hierarchy of predictors was completed by alexithymia, which, together with perceived stress, accounted for an additional part of burnout variance (up to 16% in women, for the component of emotional exhaustion). In contrast to the aforementioned variables, perceived social support played a much smaller role, contributing only to a minor proportion (2%) of the variance of depersonalization in women. This order suggests that, in medical academic contexts, to prevent and address burnout, it is crucial to prioritize (1) the alleviation of the perception of stress and/or addressing frank symptoms produced by stress (for example through individual counseling) and (2) the creation of conditions necessary for the secure expression of one’s emotions (thereby preventing alexithymia).

Gender seems to have a distinctive influence on the development of burnout in women, particularly in what concerns emotional exhaustion. According to our findings, this can be explained, at least partially, by the higher perceived stress and alexithymia in this group. Concerning perceived stress, female medical students obtained significantly higher scores than men (*p* < 0.002), regardless of having received a similar degree of perceived social support (*p* < 0.738, *ns*). With respect to alexithymia, young women obtained higher scores than young men (*p* < 0.016) (in contrast to older adults [60]). This finding is supported by other studies on this topic [61,62], which claim that young females could be more prone to alexithymia, particularly in stressful circumstances, due to poorer emotional expressivity, greater difficulty in identifying and describing their feelings, and an externally oriented thinking style. As a whole, our findings concerning the higher relative risk of burnout in women compared to men are backed up by several literature data, especially those identifying women as processing negative life events in a more emotional way [63]. The inability to identify one’s feelings in relation to a stressful circumstance or to describe them (both components of alexithymia), or the feeling of not having support in certain circumstances, seems to predispose young women to focus on the impossibility to overpass difficulties, which creates the condition for emotional exhaustion and burnout. Another possible mechanism is the overestimation of one’s own level of stress. In contrast, alexithymia and perceived social support play a much less significant role (or none) in the dynamic of burnout in men, giving rise to the hypothesis that burnout in this group is created merely by the objective elements of the stressful situation.

The limitations of our study stem mainly from its cross-sectional design and the generalizability of the results. To address this, larger prospective multicenter studies are needed. Ideally, these would evaluate students prior to the start of their studies, during medical school and then again after the completion of their degree, as young doctors. Such studies could also assess additional factors that may influence the onset of burnout (e.g., sense of coherence, optimism) or the reversed relationships between study variables.

In part, our study limitations are due to the mere heterogeneous nature of the studies regarding burnout in academic settings, which challenges our ability to collect enough comparable data or to assess its significance. In this sense, a consensus among researchers in this field could be very useful towards the creation of a gold standard for studying burnout in the academic environment, as well as a set of recommendations for “best practice.”

## 5. Conclusions

The results of the current study point out that perceived stress and alexithymia may play a significant role in the development of burnout syndrome in medical students, particularly in women. The cumulative risks for burnout brought on by these two psychological characteristics, in conjunction with the impact of other contextual environmental factors, raise the issue of addressing them in an early phase, in order to prevent the development of burnout after years of future practice [64,65]. Our findings provide a basis for a local initiative to develop a series of interventions focused on preventing burnout in medical students, similar to the established programs for practicing physicians (such as Narrative Medicine or Balint groups). Designing isomorphic programs dedicated to medical students that address perceived stress and alexithymia with regard to gender-specificity is indeed a challenging task, but it is also a strategy useful for minimizing the risk of burnout.

## Figures and Tables

**Table 1:** Gender differences.

Variables	Men (*n* = 94) M (SD)	Women (*n* = 205) M (SD)	*p* *
Burnout components			
EE	18.66 (7.87)	22.20 (9.23)	0.001
DP	12.34 (6.52)	11.01 (7.00)	0.120
LPA	18.88 (8.40)	20.24 (8.01)	0.182
Alexithymia	43.99 (8.09)	46.93 (10.47)	0.016
Perceived social support	38.55 (7.05)	38.268 (6.72)	0.738
Perceived stress	17.40 (7.08)	20.05 (6.85)	0.002

* *t*-test for independent samples (*df* = 297); M = Mean, SD = standard deviation; EE = emotional exhaustion; DP = depersonalization; LPA = low personal accomplishment.

**Table 2:** Pearson’s correlation matrix.

Variables	EE		DP		LPA	
	Men	Women	Men	Women	Men	Women
Perceived stress	0.285 *	0.386 ^§^	0.240 ^♦^	*ns*	0.503 ^§^	0.300 ^§^
Alexithymia	0.218 ^†^	0.186 ^†^	0.241 ^♦^	*ns*	*ns*	0.231 ^§^
Perceived social support	*ns*	−0.200 *	−0.222 ^†^	−0.163 ^♦^	*ns*	−0.177 *

Only correlations of the dependent variables (EE, DP and LPA) were included. EE = emotional exhaustion; DP = depersonalization; LPA = low personal accomplishment. * *p* < 0.003; ^§^ *p* < 0.0005; ^†^ *p* < 0.002; ^♦^ *p* < 0.01.

**Table 3:** Predictors of burnout components (hierarchical multiple backward regression) *.

Gender	Variables in the Model	Regression Coefficient	95% CI	Standardized Beta	*p*	*R^2^*
Emotional exhaustion						
Men	Perceived stress	0.317	0.080 ÷ 0.516	0.285	0.005	0.119
Women	Perceived stress	0.496	0.325 ÷ 0.668	0.369	0.0005	0.168
	Alexithymia	0.124	0.012 ÷ 0.237	0.141	0.030	
Depersonalization						
Men	Perceived stress	0.203	0.021 ÷ 0.385	0.221	0.029	0.106
	Alexithymia	0.178	0.019 ÷ 0.337	0.222	0.028	
Women	Perceived social support	−0.170	−0.312 ÷ −0.027	−0.163	0.020	0.027
Low personal accomplishment						
Men	Perceived stress	0.597	0.385 ÷ 0.809	0.503	0.0005	0.245
Women	Perceived stress	0.323	0.171 ÷ 0.475	0.276	0.0005	0.129
	Alexithymia	0.151	0.052 ÷ 0.251	0.198	0.003	

* Variables included in the analysis were perceived stress, alexithymia, and perceived social support. *p*-value for inclusion in the model was 0.05.

## References

[1] Radcliffe C., Lester H. (2003). Perceived stress during undergraduate medical training: A qualitative study. Med. Educ..

[2] Moffat K.J., McConnachie A., Ross S., Morrison J.M. (2004). First year medical student stress and coping in a problem-based learning medical curriculum. Med. Educ..

[3] Guthrie E., Black D., Bagalkote H., Shaw C., Campbell M., Creed F. (1998). Psychological stress and burnout in medical students: A five-year prospective longitudinal study. J. R. Soc. Med..

[4] Dahlin M., Joneborg N., Runeson B. (2005). Stress and depression among medical students: A cross-sectional study. Med. Educ..

[5] Dyrbye L.N., West C.P., Satele D., Boone S., Tan L., Sloan J., Shanafelt T.D. (2012). Burnout among U.S. medical students, residents, and early career physicians relative to the general U.S. population. Acad. Med..

[6] Dyrbye L.N., Harper W., Durning S.J., Moutier C., Thomas M.R., Massie F.S., Eacker A., Power D.V., Szydlo D.W., Sloan J.A. (2011). Patterns of distress in US medical students. Med. Teach..

[7] Heinen I., Bullinger M., Kocalevent R.D. (2017). Perceived stress in first year medical students—Associations with personal resources and emotional distress. BMC Med. Educ..

[8] Ishak W.W., Lederer S., Mandili C., Nikravesh R., Seligman L., Vasa M., Ogunyemi D., Bernstein C.A. (2009). Burnout during residency training: A literature review. J. Grad. Med. Educ..

[9] Spangler G., Pekrun R., Kramer K., Hofmann H. (2002). Student’s emotions, psychological reactions, and coping in academic exams. Anxiety Stress Coping.

[10] Maslach C., Jackson S. (1981). The measurement of experienced burnout. J. Organ. Behav..

[11] Maslach C., Schaufeli W.B., Leiter M.P. (2001). Job Burnout. Ann. Rev. Psychol..

[12] Dyrbye L.N., Thomas M.R., Massie F.S., Power D.V., Eacker A., Harper W., Durning S., Moutier C., Szydlo D.W., Novotny P.J. (2008). Burnout and suicidal ideation among U.S. medical students. Ann. Intern. Med..

[13] Santen S.A., Holt D.B., Kemp J.D., Hemphill R. (2010). Burnout in medical students: Examining the prevalence and associated factors. South. Med. J..

[14] Dahlin M.E., Runeson B. (2007). Burnout and psychiatric morbidity among medical students entering clinical training: A three year prospective questionnaire and interview-based study. BMC Med. Educ..

[15] Dyrbye L.N., Thomas M.R., Huntington J.L., Lawson K.L., Novotny P.J., Sloan J.A., Shanafelt T.D. (2006). Personal life events and medical student burnout: A multicenter study. Acad. Med..

[16] Prins J.T., Gazendam-Donofrio S.M., Tubben B.J., Van Der Heijden F.M.M.A., Van De Wiel H.B.M., Hoeskstra-Weebers J.E.H.M. (2007). Burnout in medical residents: A review. Med. Educ..

[17] Prins J.T., Hoekstra-Weebers J.E.H.M., Gazendam-Donofrio S.M., Van De Wiel H.B.M., Sprangers F., Jaspers F.C.A., van der Heijden F.M.M.A. (2007). The role of social support in burnout among Dutch medical residents. Psychol. Health Med..

[18] Jackson E.R., Shanafelt T.D., Hasan O., Satele D.V., Dyrbye L.N. (2016). Burnout and Alcohol Abuse/Dependence among U.S. Medical Students. Acad. Med..

[19] Dyrbye L.N., Thomas M.R., Power D.V., Durning S., Moutier C., Massie F.S., Harper W., Eacker A., Szydlo D.W., Sloan J.A. (2010). Burnout and serious thoughts of dropping out of medical school: A multi-institutional study. Acad. Med..

[20] Thomas M.R., Dyrbye L.N., Huntington J.L., Lawson K.L., Novotny P.J., Sloan J.A., Shanafelt T.D. (2007). How do distress and well-being relate to medical student empathy? A multicenter study. J. Gen. Intern. Med..

[21] Daly M.G., Willcock S.M. (2002). Examining stress and responses to stress in medical students and new medical graduates. Med. J. Aust..

[22] Dyrbye L.N., Thomas M.R., Huschka M.M., Lawson K.L., Novotny P.J., Sloan J.A., Shanafelt T.D. (2006). A multicenter study of burnout, depression, and quality of life in minority and nonminority US medical students. Mayo Clin. Proc..

[23] Dyrbye L.N., Thomas M.R., Shanafelt T.D. (2006). Systematic review of depression, anxiety, and other indicators of psychological distress among U.S. and Canadian medical students. Acad. Med..

[24] Fares J., Al Tabosh H., Saadeddin Z., El Mouhayyar C., Aridi H. (2016). Stress, burnout and coping strategies in preclinical medical students. N. Am. J. Med. Sci..

[25] Fang D.Z., Young C.B., Golshan S., Moutier C., Zisook S. (2012). Burnout in premedical undergraduate students. Acad. Psych..

[26] Hafferty F.W. (1998). Beyond curriculum reform: Confronting medicine’s hidden curriculum. Acad. Med..

[27] Sreeramareddy C.T., Shankar P.R., Binu V.S., Mukhopadhyay C., Ray B., Menezes R.G. (2007). Psychological morbidity, sources of stress and coping strategies among undergraduate medical students of Nepal. BMC Med. Educ..

[28] Ball S., Bax A. (2002). Self-care in medical education: Effectiveness of health-habits interventions for first-year medical students. Acad. Med..

[29] Alarcon G., Eschleman K., Bowling N. (2009). Relationships between personality variables and burnout: A meta-analysis. Work Stress.

[30] Chang E.C., Rand K.L., Strunk D.P. (2000). Optimism and risk for burnout among working college students: Stress as a mediator. Pers. Individ. Diff..

[31] Yildirim I. (2008). Relationships between burnout, sources of social support and sociodemographic variables. Soc. Behav. Pers..

[32] Sifneos P.E. (1973). The prevalence of alexithymic characteristics in psychosomatic patients. Psychother. Psychosom..

[33] Taylor G.J., Bagby R.M., Parker J.D.A. (1997). Disorders of Affect Regulation: Alexithymia in Medical and Psychiatric Illness.

[34] Kokkonen P., Karvonen J.T., Veijola J., Lasky K., Jokelainen J., Jarvelin M.R., Joukamaa M. (2001). Prevalence and sociodemographic correlates of alexithymia in a population sample of young adults. Compr. Psychiatry.

[35] Fukunishi I., Berger D., Wogan J., Kuboki T. (1999). Alexithymic traits as predictors of difficulties with adjustment in outpatient cohort of expatriates in Tokyo. Psychol. Rep..

[36] Franz M., Popp K., Schaefer R., Sitte W., Schneider C., Hardt J., Decker O., Braehler E. (2008). Alexithymia in the German general population. Soc. Psychiatry Psychiatr. Epidemiol..

[37] Larsson M.R., Bäckström M., Michel P.O., Lundh L.G. (2010). The stability of alexithymia during work in a high-stress environment: A prospective study of Swedish peacekeepers serving in Kosovo. Scand. J. Psychol..

[38] Bratis D., Tselebis A., Sikaras C., Moulou A., Giotakis K., Zoumakis E., Ilias I. (2009). Alexithymia and its association with burnout, depression and family support among Greek nursing staff. Hum. Resour. Health.

[39] Mattila A.K., Ahola K., Honkonen T., Salminen J.K., Huhtala H., Joukamaa M. (2007). Alexithymia and occupational burnout are strongly associated in working population. J. Psychosom. Res..

[40] Lazzari D., Pisanti R., Avallone F. (2006). Perception of organizational climate and burnout amongst health care workers: The role of alexithymia as a moderator. G. Ital. Med. Lav. Ergon..

[41] Lală A., Bobîrnac G., Tipa R. (2010). Stress levels, Alexithymia, Type A and Type C personality patterns in undergraduate students. J. Med. Life.

[42] Schmitz M.J. (2000). Alexithymia, self-care, and satisfaction with life in college students.. Diss. Abstr. Int..

[43] Pöhlmann K., Jonas I., Ruf S., Harzer W. (2005). Stress, burnout and health in the clinical period of dental education. Eur. J. Dent. Educ..

[44] Schaufeli W.B., Buunk B.P., Schabraq M.J., Winnubst J.A.M., Cooper C.L. (2002). Burnout: An overview of 25 years of research and theorizing. The Handbook of Work and Health Psychology.

[45] Carpenter B.N., Steffen P.R., Haas L.J. (2004). Stress. Handbook of Primary Care Psychology.

[46] Cohen S., Hoberman H.M. (1983). Positive events and social support as buffers of life change stress. J. Appl. Physiol..

[47] Heiman T., Kariv D. (2004). Relations of perceived stress and support and the experience of coping among students in higher education. Mediterr. J. Educ. Stud..

[48] Brissette I., Schreier M.F., Carver C.S. (2002). The role of optimism in social network development, coping, and psychological adjustment during a life transition. J. Pers. Soc. Psychol..

[49] Masten R., Tusak M., Zalar B., Ziherl S. (2009). Stress, coping and social support in three groups of university students. Psychiatr. Danub..

[50] Dwyer A.L., Cummings A.L. (2001). Stress, self-efficacy, social support, and coping strategies in university students. Can. J. Counsel..

[51] Bagby R.M., Parker J.D., Taylor G.J. (1994). The twenty-item Toronto Alexithymia Scale—I. Item selection and cross-validation of the factor structure. J. Psychosom. Res..

[52] Cohen S., Karmarck T., Mermelstein R. (1983). A global measure of perceived stress. J. Health Soc. Behav..

[53] Broadhead W.E., Gehlbach S.H., DeGruy F.V., Kaplan B.H. (1988). The Duke-UNC Functional Social Support Questionnaire: Measurement of social support in family medicine patients. Med. Care.

[54] Leiter M.P., Durup J. (1996). Work, home, and in-between: A longitudinal study of spillover. J. Appl. Behav. Sci..

[55] Rafferty J.P., Lemkau J.P., Purdy R.R., Rudisill J.R. (1986). Validity of the Maslach Burnout Inventory for family practice physicians. J. Clin. Psychol..

[56] Taylor G.J., Bagby R.M., Luminet O., Parker J.D.A., Bar-On R. (2000). Assessment of alexithymia: Self-report and observer-rated measures. The Handbook of Emotional Intelligence.

[57] Maslach C., Jackson S.E., Leiter M.P. (1996). Maslach Burnout Inventory Manual.

[58] Gustafsson G., Eriksson S., Strandberg G., Norberg A. (2010). Burnout and perceptions of conscience among health care personnel: A pilot study. Nurs. Ethics.

[59] Eckleberry-Hunt J., Lick D., Boura J., Hunt R., Balasubramaniam M., Mulhem E., Fisher C. (2009). An exploratory study of resident burnout and wellness. Acad. Med..

[60] Levant R.F., Hall R.J., Williams C.M., Hasan N.T. (2009). Gender differences in alexithymia. Psychol. Men Masculin..

[61] Quinton S., Wagner H.L. (2005). Alexithymia, ambivalence over emotional expression, and eating attitudes. Pers. Individ. Dif..

[62] Eizaguirre A.E., Cabezon A.O.S., Alda I.O., Olariaga L.J., Juaniz M. (2004). Alexithymia and its relationships with anxiety and depression in eating disorders. Pers. Individ. Dif..

[63] Nolen-Hoeksema S. (2012). Emotion regulation and psychopathology: The role of gender. Annu. Rev. Clin. Psychol..

[64] Cordes C.L., Dougherty T.W., Blum M. (1997). Patterns of burnout among managers and professionals: A comparison of models. J. Organ. Behav..

[65] Mazurkiewicz R., Korenstein D., Fallar R., Ripp J. (2012). The prevalence and correlations of medical student burnout in the pre-clinical years: A cross-sectional study. Psychol. Health Med..

